# Development of a Healthy Lifestyle Assessment Toolkit for the General Public

**DOI:** 10.3389/fmed.2019.00134

**Published:** 2019-06-27

**Authors:** Flávio Reis, Bebiana Sá-Moura, Diana Guardado, Patrícia Couceiro, Luis Catarino, Anabela Mota-Pinto, Manuel T. Veríssimo, Ana Maria Teixeira, Pedro L. Ferreira, Margarida P. Lima, Filipe Palavra, Luis Rama, Lelita Santos, Roel A. van der Heijden, Carlos E. Gonçalves, António Cunha, João O. Malva

**Affiliations:** ^1^Faculty of Medicine, Institute of Pharmacology & Experimental Therapeutics, University of Coimbra, Coimbra, Portugal; ^2^Faculty of Medicine, Coimbra Institute for Clinical and Biomedical Research (iCBR), University of Coimbra, Coimbra, Portugal; ^3^Laboratory of Automatics and Systems, Pedro Nunes Institute (IPN), Coimbra, Portugal; ^4^Faculty of Sport Sciences and Physical Education, Research Center for Sport and Physical Activity (CIDAF), University of Coimbra, Coimbra, Portugal; ^5^Faculty of Medicine, General Pathology Institute, University of Coimbra, Coimbra, Portugal; ^6^Faculty of Medicine, Center for Research in the Environment, Genetics and Oncobiology (CIMAGO), University of Coimbra, Coimbra, Portugal; ^7^Ageing@Coimbra, EIP on AHA Reference Site, Coimbra, Portugal; ^8^Service of Internal Medicine, University of Coimbra Hospital (CHUC), Coimbra, Portugal; ^9^Faculty of Economics, Centre for Health Studies and Research, University of Coimbra, Coimbra, Portugal; ^10^Department of Clinical Psychology, Faculty of Psychology and Educational Sciences, University of Coimbra, Coimbra, Portugal; ^11^Neuropediatrics Unit, Centre for Child Development, Pediatrics Hospital, University of Coimbra Hospital (CHUC), Coimbra, Portugal; ^12^Unit of Nutrition and Dietetics, University of Coimbra Hospital (CHUC), Coimbra, Portugal; ^13^Center for Development and Innovation (CDI), University Medical Center Groningen (UMCG), University of Groningen, Groningen, Netherlands; ^14^Healthy Ageing Network Northern Netherlands (HANNN), Groningen, Netherlands; ^15^Faculty of Sport Sciences and Physical Education, University of Coimbra, Coimbra, Portugal

**Keywords:** healthy living, active aging, lifestyle, risk factors, citizens

## Abstract

The prevalence of age-related non-communicable chronic diseases has increased worldwide, being the leading causes of morbidity and death in many world regions, including in Europe. Innovative models and strategies focused on preventive care, including early identification of risk factors underlying disease onset and progression, and proper modification of lifestyle habits and behaviors, might contribute to promote quality of life, healthy living and active aging. Healthy Lifestyle Innovative Quarters for Cities and Citizens (HeaLIQs4cities) is an EIT Health-funded project aiming to engage, empower and educate citizens toward healthy lifestyles. One of the major objectives of this project was to develop a toolkit for a rapid and informal assessment of healthy lifestyles, to be used at different levels of care pathways, including in informal public environments. In this paper, we describe the methodology underlying the development of the toolkit, which resulted from the collaboration of an interdisciplinary focus group of academic experts, from medicine, sport sciences, psychology, health economics, and innovative technologies applied to health. The following eight components were included in the toolkit: (1) anthropometric assessment and cardiometabolic parameters; (2) physical activity and exercise; (3) well-being, social cohesion, and functional independence; (4) nutrition; (5) mental health; (6) smoking, drinking, and use of illicit substances; (7) sleep habits and quality; and (8) health and disease. A traffic light rating system indicating the risk score was used (low: green; moderate: yellow; and relevant: orange) for each of the 8 components, together with recommendations for the toolkit users. After completing the reduced version of the toolkit, individuals showing moderate or relevant risk in one or more of the 8 dimensions, were invited to participate in a more detailed assessment (toolkit long version), based on deeper and scientifically validated tools. The toolkit was incorporated in eVida, a web-based platform that focuses on delivering services to personalized health and well-being. The validation of the current toolkit has been applied in wide-ranging public events in three different European Regions. Large scale deployment of the toolkit is expected to profit from the Reference Site Collaborative Network of the European Innovation Partnership on Active and Healthy Aging (EIP on AHA).

## Introduction

During the past century human life expectancy has almost doubled in several developed countries, mainly due to effective drugs, better nutritional, and hygienic/sanitary conditions, as well as improved healthcare. This phenomenon, in conjunction with decreasing birth rates in many world regions, is causing a considerable increment in the world elderly population (1). Therefore, by 2030, the population aged over 60 is expected to rise more than 50%, to over 1.4 billion people (2). Europe has been particularly affected and Portugal, with a predicted total age dependency ratio of 89.7% in 2070, will be among the highest in Europe (3). The demographic changes are especially prominent in the Center Region of Portugal, which currently shows one of the highest aging indexes in Europe (3).

People live longer but not necessarily in a healthy condition. In fact, the prevalence of age-related chronic diseases has increased in many countries, including cardiovascular diseases, as well as cancer and neurodegenerative diseases, becoming the leading causes of morbidity and death worldwide (4, 5). Another evidence is the distribution of the healthy life expectancy (6). This has a major social and economic impact due to a decrease in the working population associated with a concomitant increase in the retired one that requires healthcare and economic assistance.

Therefore, there is an urgent need to find better strategies to prevent the development of age-related diseases, or at least to slow down the rate of aging and especially functional dependency, thus improving the quality of life and reducing the burden of social and medical costs. There is an imperative need to shift our current way of managing age-related chronic disease from a system that focus on treatment of disorders, already installed, to one that concentrates on preventive and integrated care (7, 8).

The leading causes of disability and death in many world regions are non-communicable disorders, such as obesity, hypertension, dyslipidemia, stress, and anxiety, often associated with modifiable risk factors related with lifestyle habits, including hypercaloric diets, physical inactivity, as well as smoking and drinking habits. Therefore, early identification of individuals at-risk and proper modification of lifestyle habits and behaviors would have an impact on morbidity and premature mortality worldwide (6, 9), as well as on supporting long living, with functional independence and healthy aging (10, 11).

Longevity medicine has been adopting a holistic and proactive view of health in order to prevent chronic disease, with integrative evaluation of health-related habits, including those associated with disease promotion and those related with general health and well-being (12). Currently, primary care is, for most patients, the main health care system setting to begin addressing health-related habits, which is clearly insufficient due to several limitations: the first is that it is reactive since patients visit their doctor with a complaint; the second is the fact that doctors are not trained in lifestyle medicine; the third is the lack of time of both the patients and doctors; and the fourth are difficulties to simultaneously manage several risk factors (13).

In this context, innovative models, projects, and solutions that promote healthy living and active aging are required, as they can be an important stimulus for a health sustainable economy, particularly in the European regions affected by aging of the population (14), as has been supported by the European Innovation Partnership on Active and Healthy Aging (EIP on AHA) (15). The European Institute of Innovation and Technology (EIT) created a Knowledge and Innovation Community (KIC), the EIT Health, dedicated to tackling the demographic challenge posed by population aging. EIT Health drives business creation by stimulating entrepreneurship and by funding innovative initiatives that will engage, empower, and educate citizens toward healthy lifestyles. HeaLIQs4Cities is an EIT Health collaborative project encompassing the University Medical Center Groningen (UMCG), in The Netherlands, the University of Coimbra (UC) and the Instituto Pedro Nunes (IPN), in Portugal. The main aim of HeaLIQs4Cities is to join healthy lifestyles actors aligned with the concept of the EIP on AHA quadruple helix, enabling the interaction among citizens, local academia, small and medium-sized enterprises, and government at Lifestyle Innovation Quarters (LIQs) (16). In parallel with the development of health literacy and technologies applied to health and well-being, HeaLIQs4Cities main task was to develop a toolkit for a rapid and informal assessment of healthy lifestyles, to be further incorporated into different levels of health care. Previous reports suggest that this type of tools could be useful to evaluate health-related habits and lifestyle and complement current approaches with new models of care (17, 18).

Our toolkit has been equipped with validated instruments and innovative technologies, and includes components focusing on the main modifiable cardiovascular risk factors, psychological and social dimensions, mental health, as well as sleep quality (19, 20). In this report, we describe the methodology used to develop the healthy lifestyle assessment toolkit.

## Methods

### Setting

This toolkit was developed in Coimbra, Portugal (by the University of Coimbra—UC—and the Pedro Nunes Institute—IPN) in collaboration with the University Medical Center Groningen (The Netherlands) under the scope of the European project “Healthy Lifestyle Innovation Quarters for Cities and Citizens (HeaLIQs4Cities)—code 18036” funded by the EIT Health.

### Focus Group of Experts

**Figure 1:**
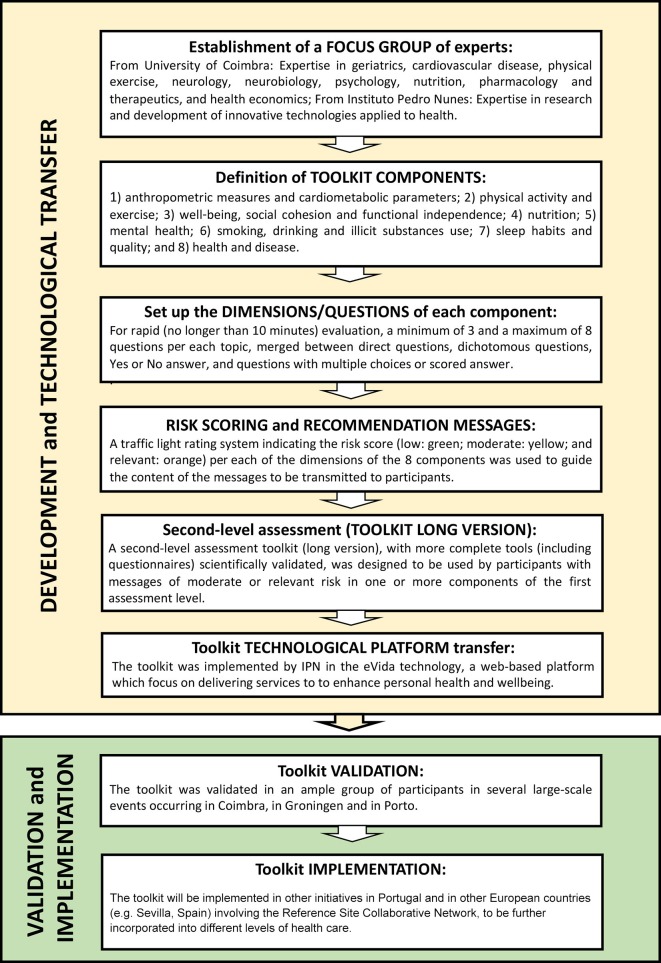
Steps of toolkit development, incorporation in the eVida technology and further validation and implementation.

The development of the toolkit to assess the perception of healthy lifestyles and risk factors began with the establishment of a Focus Group made up of academic experts in the areas of geriatrics, risk factors for cardiovascular disease, exercise, neurology, neurobiology, psychology, nutrition, pharmacology and therapeutics, health economics and innovative technologies applied to health. This Focus Group got together in a series of 4 formal meetings, followed by smaller group discussions with the objective of creating a simple, multidimensional, user-friendly toolkit to assess healthy lifestyles. In order to achieve that goal, the Focus Group decided on: (1) the dimensions of the toolkit to be assessed; (2) the instruments (technology and questionnaires) included in the toolkit and (3) the content and format of the recommendations report. Figure 1 presents the steps of the toolkit development.

### Toolkit Scope and Components

The Focus Group started by defining the components to be included in the toolkit; a first part for general biographic data, including age, gender, educational level, and current occupation, followed by 8 scientific components: (1) anthropometric assessment and cardiometabolic parameters; (2) physical activity and exercise levels; (3) well-being, social cohesion and functional independence; (4) nutrition; (5) mental health; (6) smoking, drinking and illicit substances use; (7) sleep habits and quality; (8) health and disease.

Given the aim of constructing an instrument for rapid (no longer than 10 min) evaluation of perception of healthy lifestyles covering the above described components, the focus group established that the reduced/rapid version of the toolkit should include a minimum of 3 and a maximum of 8 questions per dimension. These included several types of questions, namely: direct ones related to anthropometric or biochemical parameters, dichotomous questions, Yes or No answer, multiple choice and scaled questions.

### Risk Scoring, Messages and Second-Level Assessment

From the constructed questionnaire, which was concise and based on direct and pragmatic questions, a traffic light rating system indicating the risk score (low: green; moderate: yellow; and relevant: orange) for each of the dimensions of the 8 components was used.

A second-level assessment toolkit (long version), with more detailed and scientifically validated complex tools (including equipments and questionnaires), was developed. People receiving moderate or relevant risk scores in the first level of assessment were invited to the second-level assessment.

### Toolkit Technological Platform Transfer

The toolkit was implemented in the eVida technology platform. eVida is a web-based platform which focuses on delivering services to enhance personal health and well-being and was developed under the management of IPN. eVida allows the development and integration of applications and medical devices and is compliant with the most common standards for health-related data (e.g., HL7) and device communication (e.g., IEEE11073).

## Results

### Toolkit Questionnaire and Associated Instruments

The complete toolkit questionnaire is presented on Table 1. The preliminary information regarding general biographic data of subjects includes the following parameters: age, gender, educational level, and current occupation. No personal data allows the identification of subjects.

**Table 1:** Reduced/rapid version of the lifestyle assessment toolkit.

**Components and questions**
**General characterization data**
Age (yrs)
Gender (Male/Female)
What is the highest degree or level of school you have completed? *If currently enrolled, highest degree received. Options:* None 1st cycle of Basic Education (1st−4th year)/Old 4th class 2nd cycle of Basic Education (5th−6th year)/Old 6th grade/Preparatory Cycle 3rd cycle of Basic Education (7th−9th year)/General Course Secondary Education (10th−12th year)/Complementary Course Higher Education (Polytechnic or University) Prefer not to answer What is your professional situation? What is your employment *status*? *Options:* Unemployed Employed (or taking care of domestic tasks) Student (or doing an unpaid internship/stage) Unable to work/Disabled Retired Prefer not to answer
**1. Anthropometric measurements and cardiometabolic parameters**
1.1. Weight (in Kg); Height (in m); Body mass index (in Kg/m^2^) | *(to be measured in site)* 1.2. Waist circumference (cm) | *(to be measured in site)* 1.3. Blood pressure*:* Systolic (mmHg); Diastolic (mmHg) | *(to be measured in site)* 1.4. Glycemia (mg/dL): Normal/High/Unknown 1.5. Total cholesterol (mg/dL): Normal/High/Unknown
**2. Physical activity and exercise**
2.1. Thinking about your day-to-day life, do you consider you have a sedentary lifestyle? Yes/No 2.2.How long, on average, do you spend, per day walking? Options: <30 min/30 to 60 min/More than 60 min 2.3. Do you exercise regularly? No /Yes If Yes: Ball games Running outdoor Group lessons (yoga, pilates, etc.) Fitness equipment Swimming or water aerobics Other(s) 2.4. How many days a week do you exercise? Options: 1 or 2 days/3 or more days
**3. Well-being, social cohesion, and functional independence**
3.1. Indicate your level of agreement with the following sentence: “*I am satisfied with my life.”* Options: Strongly disagree Disagree Neither agree nor disagree Agree Strongly agree Prefer not to answer 3.2. Between family and friends, how many people could you ask for help, if needed? Options: None/1 or 2 people/3 or 5 people/6 or more 3.3. Can you perform the following day-to-day activities without limitations? (*only for people with >65 years old*) 3.3.1. Personal hygiene (washing and dressing yourself): Yes/No 3.3.2. Mobility (going up and down stairs without limitations): Yes/No 3.3.3. Dealing with money (shopping, paying bills, etc.): Yes/No/Not applicable 3.3.4. Regular use of digital technologies (computer, e-mail, smartphone, social networks, etc.): Yes/No/Not applicable
**4. Nutrition**
4.1. Do you follow a healthy and varied diet? Yes/No
4.2. In a typical week, how often do you eat/drink the following food/drinks? Options: Once or more per day/4–6 times per week/1–3 times per week/ <1 time per week/Never Vegetables and fruit Milk and milk products Fish, meat and eggs Bread, pasta or cereal Legumes (beans, peas) and Grains Fried and salty foods Sweets (desserts, soft drinks or >5 cookies)
**5. Mental health**
5.1. Has any doctor told you that you suffer from mental problems? No/Yes If Yes: Depression Anxiety and/or permanent stress Easy Irritation Obsessive-compulsive disorder Bipolar disorder Anorexia nervosa Other(s) 5.2. You have, or had, any of the following diseases? Stroke Parkinson Epilepsy Lack of memory Nausea, dizziness, migraine Other(s) No
**6. Smoking, drinking, and illicit substances use**
6.1. Do you smoke regularly? Yes/No 6.2. Did you use to smoke regularly? Yes/No 6.3. When did you quit smoking? Options: <1 year ago/1 up to 5 years ago/5 up to 10 years ago/More than 10 years ago 6.4. Are you a regular consumer of alcohol (more than one glass of wine or beer with the meals)? Yes/No 6.5. Have you ever been a regular consumer of alcoholic drinks? Yes/No 6.6. Do you use drugs regularly? Yes/No 6.7. Have you ever used drugs regularly? Yes/No
**7. Sleep habits and quality**
7.1. Do you sleep well? Yes/No 7.2. How many hours per night? Options: <5 h/From 5 to <7 h/7 or more hours 7.3. How often do you wake up at night? Options: Never or once/2 or 3 times/4 or more times 7.4. Do you wake up feeling tired? Yes/No 7.5. Do you take sleeping pills? Yes/No
**8. Health and disease** (in combination with 1)
8.1. Have you ever been told by a doctor that you have/had any of the following illnesses or chronic diseases? (which lasted more than 6 months) Heart disease (insufficiency/ischemia or angina/arrhythmia) Neuropathy (peripheral nerve disease/hands and feet) Respiratory disease (asthma/bronchitis/DPOC) Retinopathy (eye disease) Peripheral arterial disease (disease of the arteries of the legs and feet) Nephropathy (renal failure) Other(s) 8.2. How many different medications (tablets, dragees, syrups, insulin, etc.) do you take per day? Options: None or just 1/2 or 5/More than 5 8.3. Do you take regularly natural products (teas, supplements, etc.) for therapeutic purposes? Yes/No 8.4. In general, how do you evaluate your health condition? Options: Very good/Good/Reasonable/Bad/Very bad 8.5. To conclude, how would you rate your health today? Choose a number between 0 and 100, in which 0 is the worst and 100 the best you can imagine

The first of eight components related with healthy lifestyle habits and health/disease conditions have been defined as anthropometric assessment and cardiometabolic parameters. It covers parameters to be evaluated on site using appropriate equipment, such as weight and height [which allows the calculation of body mass index (BMI)], waist circumference (WC) and systolic and diastolic blood pressure (SBP and DBP, respectively). In addition, toolkit users were asked to indicate (if possible) the last known (indicating the approximate date) values of blood glucose and serum total cholesterol, which were categorized in normal, high, or unknown values.

Regarding physical activity and exercise (component 2), subjects were enquired about self-perception of active or sedentary lifestyles; daily time spent walking, regular exercise practice (and sport/recreational modalities), as well as total number of days per week spent in volunteer active physical exercise.

Concerning component 3—well-being, social cohesion and functional independence, the questionnaire includes a first question to assess the level of global satisfaction with life (well-being/quality of life), a second one regarding the number of people available to help him/her if needed (social cohesion), a third one focused on functional independence when performing day-to-day activities, without limitations, including personal hygiene, mobility and managing/administrating money and the last one regarding the ability to use digital technology

General food consumption habits were addressed in component 4 (nutrition) by questioning the self-perception of healthy diet habits, and also weekly frequency of intake of food/drinks: vegetables and fruit; milk and milk products; fish, meat and eggs; bread, pasta, or cereal; legumes (beans, peas) and grains; fried and salty foods; and sweets.

A brief assessment of mental health status (component 5) was done by asking if a mental problem, such as depression, anxiety, bipolar disorder or anorexia, among others, have been ever diagnosed by a medical doctor. Additionally, the participants were asked whether they had ever suffered any other neurological condition, such as stroke, Parkinson disease, epilepsy, among others.

In component 6, related with tobacco and alcohol habits, and illicit substances consumption, participants were asked about regular smoking, alcohol drinking, or using illicit substances, or if they had used any of them regularly in the past.

Sleep habits and quality (component 7) were evaluated through direct questions related to participant's self-perception of good (or bad) sleep, number of sleeping hours per night, number of night time wake-ups, as well as feeling rested or tired when waking up and also about the need of sleeping pills.

The reduced/rapid version of the lifestyle assessment toolkit further includes some questions regarding health and disease (component 8), such as illnesses or chronic diseases, medications, and natural products regularly consumed. Finally, the questionnaire closes with the subject's self-perception of his/her general and current day health condition.

### Risk Scoring

The traffic light type risk score (low: green; moderate: yellow; and relevant: orange) for each of the 8 components was used to guide the recommendation messages resulting from the lifestyle assessment toolkit (Table 2). As a general rule, for each dimension the risk will be low if all answers are “green,” it will be moderate if at least one of the answers is “yellow,” without any “orange,” and it will be relevant if at least one answer is “orange” (Table 2).

**Table 2:** Risk score for each of the components of the reduced/rapid version of the lifestyle assessment toolkit.

**Questions**	**Answers**	**Risk score**
**1. Anthropometric measurements and cardiometabolic parameters**		
1.1.	If BMI: 18.5 to 24.99 kg/m^2^	Low
	If BMI: <18.50 kg/m^2^	Moderate
	If BMI: from 25 to 29.99 kg/m^2^	Moderate
	If BMI: ≥30 kg/m^2^	Relevant
1.2.	Men: If WC <94|Women: If WC <80	Low
	Men: If WC 94–102|Women: If WC 80–88	Moderate
	Men: If WC > 102|Women: If WC > 88	Relevant
1.3.	If SBP <130 and DBP <85 mmHg	Low
	If SBP ≥ 130 to <140 mmHg; or DBP ≥ 85 to <90	Moderate
	If SBP ≥ 140 or DBP ≥ 90 mmHg	Relevant
1.4.	If Glycemia <110 mg/dl	Low
	If Glycemia ≥ 110 to <126 mg/dl	Moderate
	If Glycemia ≥ 126 mg/dl	Relevant
1.5.	If Total cholesterol <190 mg/dl	Low
	If Total cholesterol ≥ 190 to <240 mg/dl	Moderate
	If Total cholesterol ≥ 240 mg/dl	Relevant
**2. Physical activity and exercise**		
2.1.	No	Low
	Yes	Relevant
2.2.	More than 60 min	Low
	30–60 min	Moderate
	Less than 30 min	Relevant
2.3.	Yes	Low
	No	Relevant
2.4.	1–2	Low
	3 or more	Low
**3. Well-being, social cohesion, and functional independence**		
3.1.	Strongly disagree	Relevant
	Disagree	Relevant
	Neither agree nor disagree	Moderate
	Agree	Low
	Strongly agree	Low
	Prefer not to answer	
3.2.	None	Relevant
	1 or 2 people	Moderate
	3–5 people	Low
	6 or more	Low
3.3.1.	Yes	Low
	No	Moderate
3.3.2.	Yes	Low
	No	Moderate
3.3.3.	Yes	Low
	No	Moderate
3.3.4.	Yes	Low
	No	Low
**4. Nutrition**		
4.1.	Yes	Low
	No	Moderate
4.2.	≥4 times all the options (1) to (5) and <1 time (6) and (7)	Low
	All the other others not low and relevant risk	Moderate
	Never to options (1) to (5) or ≥4 times options (6) and (7)	Relevant
**5. Mental health**		
5.1.	No	Low
	Yes	Relevant
5.1.1.	Depression	Relevant
	Anxiety and/or Permanent Stress	Relevant
	Easy Irritation	Relevant
	Obsessive Compulsive	Relevant
	Bipolar Disease	Relevant
	Anorexia nervosa	Relevant
	Other	Relevant
5.2.	Stroke	Relevant
	Parkinson	Relevant
	Epilepsy	Relevant
	Lack of memory	Relevant
	Nausea, diziness, migraine	Relevant
	Other(s)	Relevant
	No	Low
**6. Smoking, drinking, and illicit substances use**		
6.1.	No	Low
	Yes	Relevant
6.2.	No	Low
	Yes	Moderate
6.3.	Less than 1 year ago	Relevant
	1 up to 5 years ago	Moderate
	5 to 10 years ago	Moderate
	More than 10 years ago	Low
6.4.	No	Low
	Yes	Moderate
6.5.	No	Low
	Yes	Moderate
6.6.	No	Moderate
	Yes	Relevant
6.7.	No	Low
	Yes	Relevant
**7. Sleep habits and quality**		
7.1.	Yes	Low
	No	Moderate
7.2.	Less than 5 h	Relevant
	From 5 to <7 h	Moderate
	7 or more hours	Low
7.3.	Never or once	Low
	2 or 3 times	Moderate
	4 or more times	Relevant
7.4.	Yes	Relevant
	No	Low
7.5.	Yes	Relevant
	No	Low
**8. Health and disease**		
8.1.	No	Low
	Yes	Moderate
8.2.	None or just 1	Low
	2 or 5	Moderate
	More than 5	Relevant
8.3.	Yes	Moderate
	No	Low
8.4.	Very Good	Low
	Good	Low
	Reasonable	Moderate
	Bad	Relevant
	Very bad	Relevant

Regarding the anthropometric and cardiometabolic parameters assessment (component 1), BMI and WC cut-offs were defined in agreement with the WHO standards. Low risk for BMI between 18.5 and 24.99 kg/m^2^, moderate risk for underweight (<18.5 kg/m^2^) and overweight (≥25 kg/m^2^) and relevant risk for obese ≥30 kg/m^2^). Regarding WC, low risk for men <94 cm and women <80 cm; moderate risk for men 94–102 cm and women 80–88 cm; and relevant risk for men >102 cm and women >88 cm. Concerning the blood pressure, the risk was scored as follows (in mmHg): low risk if SBP <130 and DBP <85; moderate risk if SBP ≥ 130 to <140 or DBP ≥ 85 to <90 and relevant risk if SBP ≥ 140 or DBP ≥ 90. Risk related with glycemia levels (in mg/dl) was set according to the following: low risk for glycemia <110; moderate risk between 110 and <126 and relevant risk for values ≥126. For total cholesterol levels (in mg/dl), risk was defined as follows: low risk for <190; moderate risk for values between 190 to <240 and relevant risk ≥240.

Concerning physical activity and exercise (component 2), low risk was set for subjects indicating active lifestyle, more than 60 min of daily walking or practice of regular exercise, while the opposite (sedentary lifestyle, <30 min of daily walking or absence of regular exercise practice) was indicated as relevant risk. Intermediate risk was set for people without regular exercise practice but that spent 30–60 min walking daily.

In relation to well-being, social cohesion and functional independence (component 3), the risk was defined for each answer to each question. An “Agree” or “Strongly agree” with the sentence “I am satisfied with my life” was quoted as low risk, and “Neither agree nor disagree” as moderate risk and an “Disagree” or “Strongly disagree” as relevant risk. Concerning social cohesion, reporting “3 or 5” and “6 or more” people supporting him/her if needed were indicative of low risk, while reporting “1 or 2” persons was taken as moderate risk and no support was indicative of relevant risk. Regarding functional independence, inability or ability to perform day-to-day activities without limitations, including personal hygiene, mobility and dealing with money, was set as moderate or low risk, respectively.

Concerning nutrition, the fourth component of the toolkit, a personnel perception of a healthy and varied diet practice was set as low risk and the opposite as moderate risk. A low, moderate or relevant risk was also attributed considering the weekly frequency (once or more per day; 4–6 times per week; 1–3 times per week; <1 time per week or never) of eating different food/drinks, as follows: (1) vegetables and fruits; (2) milk and milk products; (3) fish, meat and eggs; (4) bread, pasta or cereal; (5) legumes (beans, peas) and grains; (6) fried and salty food; (7) sweets. Low risk was attributed when participants reported that they consume 4 or more times per week all the options (1) to (5) and consume <1 time a week the options (6) and (7). Relevant risk was attributed when participants report that never consume any of the options (1) to (5) or consume 4 or more times per week the options (6) and (7). The other intermediate answers were set as moderate risk. Special dietary habits related with religion or vegetarianism/veganism have been taken as exceptions.

Regarding the mental health (component 5), a “NO” answer to the two questions was set as low risk, while “YES” to any of the listed mental problems or neurological diseases was classified as relevant risk, deserving medical supervision and follow-up.

For component 6 (smoking, drinking, and illicit substances use), a low risk was identified for participants reporting no current smoking habits (or stopped smoking more than 10 years ago) and no regular consumption of alcohol (defined as more than one glass of wine or beer at meals) and illicit substances. Subjects reporting having stopped smoking <10 years ago were classified to be at moderate risk; also at moderate risk were subjects mentioning regular alcohol consumption or past habits of illicit substances use. Relevant risk was set for those referring current smoking habits or interruption for <1 year ago and/or current illicit substances use.

Concerning component 7 (sleep habits and quality), a low risk was attributed to people reporting perception of good sleep, lasting for at least 7 h, without taking pills and waking rested. Participants reporting <5 h of sleep time, waking up multiple times during night (4 or more), needing to take pills to sleep and the feeling of waking up tired were classified as being at relevant risk levels. Moderate risk, was defined for all the other conditions of moderate sleep time and waking up during the night.

Regarding component 8 (health and disease), a low risk was attributed to people without diagnosis of any of the diseases listed in question 8.1, not taking more than one medication and not taking regularly natural products (teas, supplements, etc.) for therapeutic purposes, as well as with self-perception of very good or good general health condition; a moderate risk was set to participants reporting previous diagnosis of any of the list disorders, or people taking between 2 and 5 different medications, or taking regularly natural products for therapeutic purposes, or reporting a self-perception of a moderate/fair general health condition; a relevant risk was attributed to people that usually take more than 5 different medications or report self-perception of health condition as bad or very bad.

### Recommendation Messages

Following completion of the reduced version of the toolkit, participants are given a report with recommendation messages regarding their lifestyle habits and associated healthy/unhealthy conditions for each one of the 8 dimensions assessed (Table 3). The messages, in agreement with the traffic light type risk score, have been categorized between low risk recommendations, typically reinforcing the need to monitor and maintain the good habits and the healthy condition, moderate risk messages addressing the need of some changes of habits in order to improve the health condition and relevant risk messages, recommending deeper change, under professional guidance, to significantly improve the parameters in the orange answers.

**Table 3:** Recommendation messages according to the risk score obtained for each dimension of the components of the reduced/rapid version of the lifestyle assessment toolkit.

	**Risk score**	**Recommendation message**
**1. Anthropometric measurements and cardiometabolic parameters**		
**BMI/WC**	Low	BMI from 18.5 to 24.99 kg/m^2^|WC <94 cm for men or <80 cm for women: Your weight is within normal range for your height, as it is your WC. Maintain a healthy lifestyle, including a balanced diet and moderate and regular physical activity, and watch for any changes in your BMI
	Moderate	BMI <18.50 kg/m^2^ For your height your weight is below normal. BMI values below those recommended may be associated with risk situations that must be followed up. We advise evaluation by doctor/nutritionist BMI from 25 to 29.99 kg/m^2^|WC from 94 to 102 cm for men or from 80 to 88 cm for women: For your height your weight is slightly increased. Being overweight is a risk factor for cardiovascular and metabolic diseases, such as diabetes. We recommend reviewing your lifestyle, including promoting a balanced diet and moderate and regular physical activity
	Relevant	BMI ≥ 30 kg/m^2^|WC > 102 cm for men and >88 cm for women: For your height your weight is clearly increased. Overweight and obesity are risk factors for cardiovascular and metabolic diseases, such as diabetes. It's time to do something to reverse this condition! We advise you to change your lifestyle, including promoting a balanced and healthier diet and moderate and regular physical activity. We suggest advice and follow-up by a doctor/nutritionist
**Blood pressure**	Low	SBP <130 mmHg and DBP <85 mmHg: Your blood pressure is normal. Maintain a healthy lifestyle, including a balanced diet and moderate and regular physical activity. In particular, do not overeat, eat fruits and vegetables, do not drink too much alcohol, and keep your weight under control
	Moderate	SBP ≥ 130 and <140 mmHg; DBP ≥ 85 and <90 mmHg: Your blood pressure is at the upper limit of normal. High blood pressure values are a risk factor for cardiovascular disease. We recommend reviewing your eating habits and physical activity. In particular, reduce salt intake, regularly eat fruits and vegetables, do not drink alcoholic beverages in excess and keep weight controlled
	Relevant	SBP ≥ 140 mmHg and DBP ≥ 90 mmHg: Your blood pressure is high. High blood pressure is a risk factor for cardiovascular disease. It's time to do something to reverse this condition! We advise you to change your lifestyle, including promoting a balanced and healthier diet and moderate and regular physical activity. In particular, reduce salt intake, regularly eat fruits and vegetables, do not drink alcoholic beverages in excess, keep weight controlled. We suggest advice and medical follow-up
**Glycemia**	Low	Glycemia <110 mg/dl: Your blood glucose is normal. Maintain a healthy lifestyle, including a balanced diet and moderate and regular physical activity. In particular, do not consume excess sugar and sweets, and keep your weight under control
	Moderate	Glycemia ≥ 110 and <126 mg/dl: Your blood glucose is slightly increased. Elevated blood glucose values are a risk factor for the development of diabetes and cardiovascular disease. We recommend reviewing your eating habits and physical activity. In particular, do not consume excess sugar and sweets, and keep your weight under control
	Relevant	Glycemia ≥ 126 mg/dl: Your blood sugar is high. Diabetes is associated with the development of various vascular diseases and complications with serious implications in your life. It's time to do something to reverse this condition! We advise you to change your lifestyle, including promoting a balanced and healthier diet and moderate and regular physical activity. We suggest advice and follow-up by a doctor
**Total Cholesterol**	Low	Cholesterol <190 mg/dl (in those who have no history of cardiovascular disease): Your cholesterol values are normal. Maintain a healthy lifestyle, including a balanced diet and moderate and regular physical activity
	Moderate	Cholesterol ≥ 190 and <240 mg/dl: Your cholesterol values are increased. High cholesterol values are a risk factor for the development of cardiovascular diseases. We recommend reviewing your eating habits and physical activity. In particular, restrict the consumption of foods high in cholesterol and animal fats, and keep weight controlled
	Relevant	Cholesterol ≥ 240 mg/dl: Your cholesterol values are greatly increased. Hypercholesterolemia is associated with a high risk of development of several diseases and vascular complications with serious implications in their life. It's time to do something to reverse this condition! We advise you to change your lifestyle, including promoting a balanced and healthier diet and moderate and regular physical activity. In particular, restrict the consumption of foods high in cholesterol and animal fats, and keep weight controlled. We suggest advice and follow-up by a doctor
**2. Physical activity and exercise**		
	Low	Your level of physical activity is above the population average. Maintain your good daily habits in order to continue increasing the number of benefits that contribute to your general level of life quality, while decreasing, at the same time, the risk of cardiovascular disease and limitations in daily functionality. If you still do not, try to challenge yourself and increase the intensity of your physical activities, such as workouts with greater cardiovascular responses, whenever possible
	Moderate	Your level of physical activity is considered moderate. Although you're being able to maintain a reasonable level of physical activity habits, you may seek to increase them in intensity or duration by performing cardiovascular exercises with greater intensity and/or by increasing the daily time spent on light activities such as walking. In this way you will increase your general level of life quality, reducing the risk of cardiovascular diseases and limitations in daily functionality
	Relevant	Your level of physical activity is below the mark that is advised for general population, being considered a risk zone. Try to accumulate a minimum of 30 min of moderate physical activity a day, or participate in more demanding cardiovascular exercises at least 3 times a week, seeking to meet daily habits that will contribute to increase your general level of life quality and to decrease the risk of cardiovascular disease and limitations in daily functionality. We advise evaluation by a specialized professional
**3. Well-being, social cohesion, and functional independence**		
	Low	Your level of well-being/quality of life is above average. Continue to value what you have in your life, to live and make friends, to join new activities and to maintain their independence and autonomy
	Moderate	Your level of well-being/quality of life is considered reasonable. Although you succeeded in living a satisfactory life, you will benefit by increasing your social and family interaction, by paying more attention to the way you think and carry out more activities that promote your happiness and sense of life and that preserve your independence and more autonomy.
	Relevant	Your level of well-being/quality of life inspires concern. Try to value yourself more and the things you have in your life, give more time to your family and friends. Make more friends. Start new activities that interest you and help you make more sense in your life. Preserve your autonomy and independence. We advise evaluation by a specialized professional
**4. Nutrition**		
	Low	Your eating habits are healthy. Maintain a varied diet based on vegetables and fruits, using daily food from all groups. Avoid fried and salted as well as alcoholic beverages. These habits will allow you to reduce the risk of chronic diseases and maintain the quality of life
	Moderate	Your eating habits need little adjustment or are not wholesome. Try to make a more varied diet and increase your consumption of vegetables and fruits, without excess of food of animal origin, although using daily foods of all groups. Avoid fried and salted as well as alcoholic beverages. These habits will allow you to reduce the risk of chronic diseases and maintain a good quality of life
	Relevant	Your eating habits have enough deficiencies compared to the recommended for the healthy population. Try to make a more varied diet and increase your consumption of vegetables and fruits, without excess of food of animal origin, although using daily foods of all groups. Avoid fried and salted as well as alcoholic beverages. These habits will allow you to reduce the risk of chronic diseases and maintain a good quality of life. We advise evaluation by doctor/nutritionist
**5. Mental health**		
	Low	Your mental health level is satisfactory. Stay active, exercise your brain and enjoy your abilities
	Relevant	Your level of mental health inspires concern. Day-to-day life can generate some difficulties, which can have a very negative impact on your emotional well-being, the health of your brain and, as a consequence, of your whole body. Try to find leisure moments, fight the sedentary lifestyle and keep the brain busy with challenging activities, exercising it as if it were a muscle. We advise specialized medical evaluation
**6. Smoking, drinking, and illicit substances use**		
**Smoking**	Low	Smokers (even if in the recent past) are exposed to high and unnecessary risk of developing various diseases, including cardiovascular, respiratory and oncological diseases, among others. For your health, maintain your non-smoker status
	Moderate	Being a regular ex-smoker only a few years (<10 years) means that you can maintain some increased risk of developing various diseases compared to a non-smoker of the same age. It is therefore vital that you remain a non-smoker to further reduce the risk
	Relevant	The fact that you are a regular smoker (or former regular smoker who has stopped for <1 year) means that you have a risk of developing a variety of diseases, including cardiovascular, respiratory and oncology, among others, compared to a non-smoker of the same age. It's time to do something to reverse this condition! Quit smoking. We suggest advice and specialized medical follow-up
**Drinking**	Low	Consumption of non-distilled alcoholic beverages (such as wine and beer), if done moderately, is not in itself an added risk factor for disease development. Keep yourself as an occasional consumer, not exceeding what is considered moderate, and watch for other important risk factors as well. Pay attention to your doctor's recommendations about alcohol consumption in drug interactions or disease-related complications
	Moderate	Being a regular consumer of alcoholic beverages (or former regular consumer) means that you may have an increased risk of developing various diseases compared to an occasional and moderate consumer of similar age. It's time to do something to reverse this condition! If necessary, look for advice and support
**Illicit substances**	Low risk	Keep yourself in this condition as a non-consumer of illicit substances, whose regular consumption is associated with an increased risk of developing various diseases, including neurodegenerative and psychiatric
	Relevant	Being a regular consumer of illicit substances (or a former regular consumer) means that you may have an increased risk of developing various diseases compared to a non-consumer of comparable age. The regular consumption of these substances is associated with an increased risk of development of several diseases, including neurodegenerative and psychiatric. It's time to do something to reverse this condition! Seek expert advice and support
**7. Sleep habits and quality**		
	Low	A sleep that allows you to sleep well, for a recommended number of hours, without taking medication, and wake up without fatigue, reduces your risk of developing various diseases associated with “bad” sleep, including cardiovascular, metabolic and respiratory, among others. Maintain this condition and watch for other important risk factors
	Moderate	Apparently your sleep is not ideal, either because it does not allow you to sleep well and wake up without fatigue, either because you sleep a few hours, or even because you need sleeping pills. This situation is somewhat worrisome, as it may not be adequately resting, but may have an increased risk of development of various diseases associated with “bad” sleep, including cardiovascular, metabolic and respiratory, among others. Thus, it is vital that you take the appropriate steps to achieve better sleep. If necessary, consult a qualified professional
	Relevant	Your sleep is far from ideal. He does not sleep well or sleep very little, and he wakes up tired, or can only sleep well on regular medication. This situation is of concern since, in addition to not resting properly, it may have an increased risk of developing several diseases associated with “bad” sleep, including cardiovascular, metabolic and respiratory, among others. It's time to do something to reverse this condition! We suggest advice and specialized medical follow-up
**8. Health and disease**		
	Low	To maintain a good level of health and well-being it is important to preserve a healthy lifestyle based on a balanced diet, regular physical and mental activity and strengthening of social relations, thus maintaining your self-confidence, independence and autonomy
	Moderate	Do not neglect your health and well-being by promoting a healthy lifestyle based on a balanced diet, regular physical and mental activity, and strengthening social relationships. Be aware of possible imbalances related to your clinical situation and/or arising from polymedication
	Relevant	It is very important to be alert to signs of discomfort. Review, and correct if necessary, your eating habits, practice of physical activity, and sociability. Be very aware of possible imbalances related to taking various medications (polymedication) and consult a doctor regularly to promote any rectifications as necessary

### Second-Level Assessment Toolkit (Long Version)

Those participants that received messages of moderate or relevant risk in any of the 8 components of the toolkit reduced version, the EQ-5D-5L questionnaire (21) and a more complete assessment with scientifically validated tools (including questionnaires and technology) was suggested for a more detailed analysis (Table 4).

**Table 4:** Questionnaires and instruments of the second-level lifestyle assessment (toolkit long version) recommended for those with a moderate or relevant risk after completing the reduced toolkit version.

**Toolkit components**	**Suggested questionnaires and instruments**
1) Anthropometric measurements and cardiometabolic parameters	•Body composition analysis in a bio-impedance weighing scale and a •More complete questionnaire regarding nutrition
2) Physical activity and exercise	•Handgrip strength test; •Body balance test; •Sit and reach test; •Reaction time test; •Heart rate variability (HRV) test
3) Well-being, social cohesion and Functional independence	•3.1 (well-being): Rosenberg Self-Esteem Scale (RSES) and/or, as optional questionnaire (only for academic purposes), the NEO Five-Factor Inventory (NEO-FFI); •3.2 (social cohesion): Social Provision Scale (SPS); •3.3 (functional independence): 12-item health survey (SF-12)
4) Nutrition	•Body composition analysis in a bio-impedance weighing scale and a •More complete questionnaire regarding nutrition
5) Mental health	•Depression: Hospital Anxiety and Depression Scale (HADS) for all participants and 30-item Geriatric Depression Scale (GDS-30) for people >65 years old; •Anxiety and/or permanent stress: Perceived Stress Scale (PSS); •Easy irritation, obsessive-compulsive disorder, bipolar disorder, anorexia nervosa or any other mental problem): RSES as optional questionnaire (only for academic purposes); •Lack of memory: Mini Mental State Examination (MMSE); •Nausea, dizziness or migraine: Migraine Disability Assessment (MIDAS)
6) Smoking, drinking, and illicit substances use	•More complete questionnaire
7) Sleep habits and quality	•Epworth Sleepiness Scale (ESS) and •Pitsburg Sleep Quality Index (PSQI)
8) Health and disease	•12-item health survey (SF-12) and/or •Spirometry and oximetry tests for respiratory problems

Regarding components 1 (anthropometric assessment and cardiometabolic parameters) and 4 (nutrition), participants with either moderate or relevant risk received suggestion of further evaluation of their condition, including body composition in a bio-impedance weighing scale (InBody, MC-780 MA, Tanita Corporation of America, Inc., USA) and a more complete questionnaire regarding nutrition habits.

Concerning component 2 (physical activity and exercise), levels of moderate or relevant risk in the reduced toolkit version could be further analyzed using adequate technologies: handgrip strength Hand Dynamometer 5000 intelligent type (THP^2^ Europe Total Health and Performance Plan, The Netherlands); body balance using a pressure plate with visual feedback (PhysioSensing, Sensing Future Technologies, Portugal); sit and reach using Sit and Reach 5000 intelligent type (THP^2^ Europe Total Health & Performance Plan, The Netherlands); reaction time using Reaction Time 5000 intelligent type (THP^2^ Europe Total Health and Performance Plan, The Netherlands); heart rate variability (HRV) using SA-3000P (Medicor®, South Korea).

Concerning component 3 (well-being, social cohesion and functional independence), distinct questionnaires were proposed for those receiving yellow or orange (moderate or relevant) risk messages in the 3 questions: 3.1 (well-being)—Rosenberg Self-Esteem Scale (RSES) (22, 23) and/or, as optional questionnaire (only for academic purposes), the NEO Five-Factor Inventory (NEO-FFI) (24); 3.2 (social cohesion)—Social Provision Scale (SPS) (25); 3.3 (functional independence)—12-item health survey (SF-12) (26).

In component 5 (mental health), participants reporting suffering from a mental problem (previously diagnosed by a medical doctor) or having a neurological disorder are directed to the following questionnaires: for depression—Hospital Anxiety and Depression Scale (HADS) (27) for all participants, and the 30-item Geriatric Depression Scale (GDS-30) for people >65 years old (28); for anxiety and/or permanent stress—Perceived Stress Scale (PSS) (29); for easy irritation, obsessive-compulsive disorder, bipolar disorder, anorexia nervosa, or any other mental problem—RSES as optional questionnaire (only for academic purposes); for subjective complaints of memory—Mini Mental State Examination (MMSE) (30); for migraine, nausea and dizziness—Migraine Disability Assessment (MIDAS) (31).

For component 6 (smoking, drinking and illicit substances use), participants with either moderate or relevant risk received suggestion of complete a more detailed questionnaire.

Regarding sleep difficulties or disorders (component 7), two additional questionnaires were offered to those at moderate or at relevant risk: the Epworth Sleepiness Scale (ESS) (32) and the Pittsburg Sleep Quality Index (PSQI) (33).

For those participants that received yellow or orange risk (moderate or relevant) messages in component 8 (health and disease) the SF-12 questionnaire was offered (26) and for those reporting respiratory problems/difficulties, it was suggested to perform the spirometry (MIR Spirobank II, Medical International Research, USA) and oximetry (Onyxii9560, Nonin Medical Inc., USA) tests.

## Discussion

The main goal of the presently described toolkit for lifestyle assessment was to develop a user-friendly instrument for a rapid and non-intrusive evaluation of lifestyles in public spaces. The toolkit was firstly developed and tested in the EIT Health funded public event Praça Vida+ (16), profiting from the organization of a major university sport competition, the EUSA Games in Coimbra (July 15–28, 2018).

The toolkit has been designed to incorporate the following key characteristics: (i) user-friendly, short-version that should not take more than 10 min to complete; (ii) anonymous, no personal data allowing identification of the user was collected; (iii) automatic data extraction and recording, with innovative technologies incorporated in the work flow of the toolkit; (iv) provide the user with a report and a summary of recommendations.

The Focus Group consensus decision led to an 8 dimensions-based toolkit, encompassing the key determinants of health and well-being considered to have critical impact in quality of life and functional independence (34): (1) anthropometric assessment and cardiometabolic parameters; (2) physical activity and exercise levels; (3) well-being, social cohesion and functional independence; (4) nutrition; (5) mental health; (6) smoking, drinking and illicit substances use; (7) sleep habits and quality; (8) health and disease.

To evaluate the overweight status, the body mass index (BMI) was used as a parameter which, due to its easy application and reliability, is today the most used anthropometric assessment method. The hypothesis of using a simple scale or the use of bio-impedance was raised; however, in order to keep the toolkit rapid and user-friendly, it was concluded that BMI provided enough information for an initial approach. BMI assessment was complemented with waist circumference measurement, which is a simple and adequate method and a good measure of visceral adiposity; together with BMI, WC is a reliable marker of obesity-related cardiometabolic risk (35), particularly in obese diabetic patients (36).

The cardiovascular risk assessment was evaluated by collecting data related to blood pressure, cholesterolemia, and glycemia, allowing a good general analysis of major factors underlying hypertension, dyslipidemia/atherosclerosis and diabetes, which are highly prevalent and incident chronic cardiometabolic diseases worldwide, including in Europe and particularly in Portugal (37, 38). Other reports addressed the development of toolkits, including the Wakeup toolkit for health professional and patients suffering from pre-diabetic conditions (39).

Physical activity and exercise levels were assessed by simple questions to report the general physical activity level of the citizen. A program of regular exercise that includes cardiorespiratory, resistance, flexibility, and neuromotor exercise training beyond activities of daily living to improve and maintain physical fitness and health is essential for most adults. The American College of Sports Medicine recommends a minimum of 30 min of daily moderate-intensity cardiorespiratory exercise training for ≥30 min per day on ≥5 days per week for a total of ≥150 min per week; vigorous-intensity cardiorespiratory exercise training for ≥20 min per day on ≥3 days per week (≥75 min per week); or a combination of moderate- and vigorous-intensity exercise to achieve a total energy expenditure of ≥500–1,000 of metabolic equivalents (MET)·min·wk in order to enjoy health benefits (40). Physical activity at all ages is now recognized as a major determinant of general health, including physical function and fitness. It is a key in preventing physical and cognitive frailty and metabolic diseases onset and progression (41).

The choice of instruments for assessing health and well-being is delicate due to the panoply of related constructs (e.g., happiness, quality of life, well-being, mental health as well as health and disease components) and the variety of scales and instruments available. In addition, it is difficult to select short and validated instruments that could be used with the heterogeneous population that was our target.

Although the majority of studies have examined how particular objective variables influence well-being, almost a century of research suggests that demographic variables, objective circumstances and life events correlate less strongly with happiness than we would expect (42). These findings lead us to consider the importance of subjective processes in happiness. Researchers within the subjectivist tradition have generally relied on self-reports (43) and the literature points that most individuals are capable of reporting on the extent to which they are a happy person (or an unhappy one) or a healthy (or unhealthy) person. The most widely used measure of happiness is the Affect Balance Scale (44), which assesses the balance of positive and negative affect experienced during the past weeks. This scale intends to be a measure of the affective component of subjective well-being. As a complement, the cognitive component of subjective well-being, has been assessed with life satisfaction scales, like Satisfaction With Life Scale (SWLS) (45). Other measures of well-being include single-item scales, such as Bradburn's (44) Global Happiness Item. Single-item global evaluations, although having the problem of not being conducive to testing psychometric properties, have many practical advantages and that was the reason for including them. Regarding functional independence, the toolkit focus on the ability to perform day-to-day activities without limitations, including personal hygiene, mobility, and managing/administrating money, as well as capacity to use digital technology. In our modern technological society, it is becoming increasingly important to consider elements reflecting the engagement of older people with digital technology, an issue traditionally missing from previous assessment tools that we have considered.

Mental health terminology, instead of cognitive health, was used as generic name in the toolkit to be more easily understood by the expected target population, with particular demographic and anthropological characteristics, thus avoiding misinterpretations that could give rise to a number of false positives or negatives that would detract from the main purpose of the toolkit. However, the instrument, particularly in the larger version, includes several tests directed to the measurement of variables related to mental health, including cognition/lack of memory (namely the Mini Mental State Examination—MMSE).The general health status, as well as sleep habits and quality, are routinely addressed, and used in our toolkit, by simple and objective widely-used questionnaires including EQ-5D-5L (21), self-reported drugs consumption or self-perception of sleep quality (32, 33) as well as general health status and drug intake, including polypharmacy conditions (46).

General information about nutrition habits was assessed using a questionnaire enclosing short and general questions for the sake of time and also because the questionnaire was designed to reach the general population. Our questionnaire encompasses nutritional and social habits determinants, taking in consideration key components of a systematic review that highlighted malnutrition, including imbalanced food consumption, impacted by health and well-being modifiable determinants, including oral, psychosocial, medication and care, health, physical function, lifestyle, eating (47).

The presently described toolkit aims to promote general public awareness about the need to support healthy choices and healthy lifestyles, investing in disease prevention and supporting healthy living:

- There is evidence that behavior changes are ignited mainly by a complex cocktail of perceived benefits than health alone, and many programs that aim to promote behavioral change have failed because of their narrow focus on specific and isolated aspects;
- Healthy lifestyles promotion must result from the joint commitment of a broad coalition of public agencies and the civil society;
- All actions must be tailored according to the needs of real people and to the social and personal environment; one of the main tasks of the focus group was to translate theoretical assumptions about health and lifestyles into measures and recommendations that fit the individual;
- Social support and practical advice should be provided and this was a major concern in the toolkit design, because the social environment is important and usually undervalued;
- The same is true for feelings, emotions, social role, and identity that are known to play a central part in behavior change.

Behavior changes are individually and socially-based needs and the requirements for change management are complex in nature. So, providing accurate information and recommendations is important, but not enough; the design of our toolkit included follow-up activities that tried to keep alive the personal interaction between the professionals and the citizens and foster patient's and citizen's empowerment (48).

The outcome of the development of the lifestyle assessment toolkit consisted in a user-friendly 8 dimension-based questionnaire instrumentalized with technology for e-health extraction data, a powerful tool for citizen engagement toward e-health approaches. Our toolkit development process is comparable with other development processes, including those involved in the eVITAL toolkit (17, 18) or the support to decision making and management toolkit e-HIT (49), among others. The benefits of incorporating non-intrusive, user-friendly technologies for data extraction and reporting encompasses among others psychological empathy/attraction by the users and identification of innovative technologies to support innovation and business targeting the citizens in socio-economic local ecosystems (50, 51).

## Data Availability

The raw data supporting the conclusions of this manuscript will be made available by the authors, without undue reservation, to any qualified researcher

## Author Contributions

FR: coordination of the Focus Group, writing the manuscript, editing figures and tables, and manuscript correction. BS-M: participation in the Focus Group, toolkit validation, and contribution to manuscript final version. DG: participation in the Focus Group, toolkit technological transfer, and contribution to manuscript final version. PC and LC: participation in the Focus Group and toolkit validation. AM-P, MV, AT, PF, ML, FP, LR, and LS: participation in the Focus Group and contribution to manuscript final version. RH: contribution to toolkit final version. CG, AC, and JM: project coordination, participation in the Focus Group and contribution to manuscript final version.

### Conflict of Interest Statement

The authors declare that the research was conducted in the absence of any commercial or financial relationships that could be construed as a potential conflict of interest.
